# Amniotic Fluid Embolism With Maternal and Neonatal Survival in the Setting of Previously Undiagnosed Placenta Accreta Spectrum 3D: A Case Report

**DOI:** 10.7759/cureus.113889

**Published:** 2026-08-03

**Authors:** Dan Schaefer, Melissa A Beaty, Stephanie Chalifour, Jennifer A Braverman, Bianca Christensen

**Affiliations:** 1 Hospital Medicine, University of Colorado Health, Fort Collins, USA; 2 Obstetrics and Gynecology, University of Colorado School of Medicine, Aurora, USA; 3 Obstetrics and Gynecology, Poudre Valley Hospital, Fort Collins, USA; 4 Pathology, Summit Pathology, Loveland, USA

**Keywords:** amniotic fluid embolism, cesarean hysterectomy, disseminated intravascular coagulation, massive transfusion protocol, maternal cardiac arrest, maternal survival, obstetric hemorrhage, placenta accreta spectrum, placenta percreta, placenta previa

## Abstract

Amniotic fluid embolism (AFE) is a rare and life-threatening obstetric emergency characterized by sudden cardiopulmonary collapse and coagulopathy. Diagnosis remains clinical, and one of exclusion, as presentation may overlap with other causes of maternal cardiovascular collapse and hemorrhage. Here we present a 34-year-old woman, gravida 7 para 2133, at 33 weeks and one day gestation with known complete placenta previa who presented with vaginal bleeding and painful uterine contractions. Ultrasound performed two weeks prior demonstrated complete placenta previa without sonographic evidence of placenta accreta spectrum (PAS). Following cesarean delivery, the patient developed acute respiratory distress and pulseless electrical activity (PEA) arrest requiring cardiopulmonary resuscitation with return of spontaneous circulation after approximately 10 minutes. Massive transfusion protocol was initiated, and cesarean hysterectomy was performed after the identification of an abnormally adherent placenta. Final pathology demonstrated PAS grade 3D (previously known as placenta percreta), a condition associated with an increased risk of AFE, with extensive myometrial invasion and serosal disruption. The operative course was further complicated by disseminated intravascular coagulopathy, massive hemorrhage, acute respiratory distress syndrome, and severe urologic injury. Despite substantial postoperative morbidity requiring later urinary diversion surgery, both maternal and neonatal survival were ultimately achieved. This case highlights the diagnostic complexity of AFE occurring concurrently with catastrophic hemorrhage from previously undiagnosed PAS and emphasizes the importance of rapid multidisciplinary management and evidence-based protocols in optimizing outcomes.

## Introduction

Amniotic fluid embolism (AFE) is a rare but life-threatening obstetric emergency characterized by sudden cardiovascular collapse, respiratory failure, altered mental status, and coagulopathy [1]. Although uncommon, with an estimated incidence of one in 8,000 to one in 80,000 deliveries, AFE remains an important cause of maternal morbidity and mortality worldwide [2].

The diagnosis of AFE remains clinical, and one of exclusion, as no definitive confirmatory test exists and presentation may overlap with other causes of maternal collapse and obstetric hemorrhage [3,4]. Management is primarily supportive, focusing on rapid hemodynamic stabilization, respiratory support, correction of coagulopathy, and coordinated multidisciplinary intervention [4].

Presented here is a case of AFE complicated by previously undiagnosed placenta accreta spectrum (PAS) grade 3D in which both maternal and neonatal survival were achieved, highlighting the importance of early recognition and prompt multidisciplinary management and evidence-based protocols in optimizing outcomes.

## Case presentation

A 34-year-old woman, G7P2133, with known complete placenta previa, at 33 weeks and one day gestation by last menstrual period consistent with six-week ultrasound presented to the obstetrical emergency department with vaginal bleeding and painful uterine contractions. The patient reported contractions beginning approximately six hours prior to presentation followed by a large-volume “gush” of vaginal bleeding approximately two hours later. Initial vital signs included a temperature of 37°C, heart rate at 102 beats/minute, respiratory rate at 18 breaths/minute, blood pressure at 104/68 mmHg, and oxygen saturation 99% on room air. Physical examination demonstrated moderate uterine contractions to palpation and blood pooling within the vaginal vault without clots, consistent with moderate active bleeding. On arrival, complete blood count (CBC) demonstrated a baseline hemoglobin of 12.8 g/dL, hematocrit of 40.3%, and platelet count of 150x10^9^/L. 

**Figure 1:**
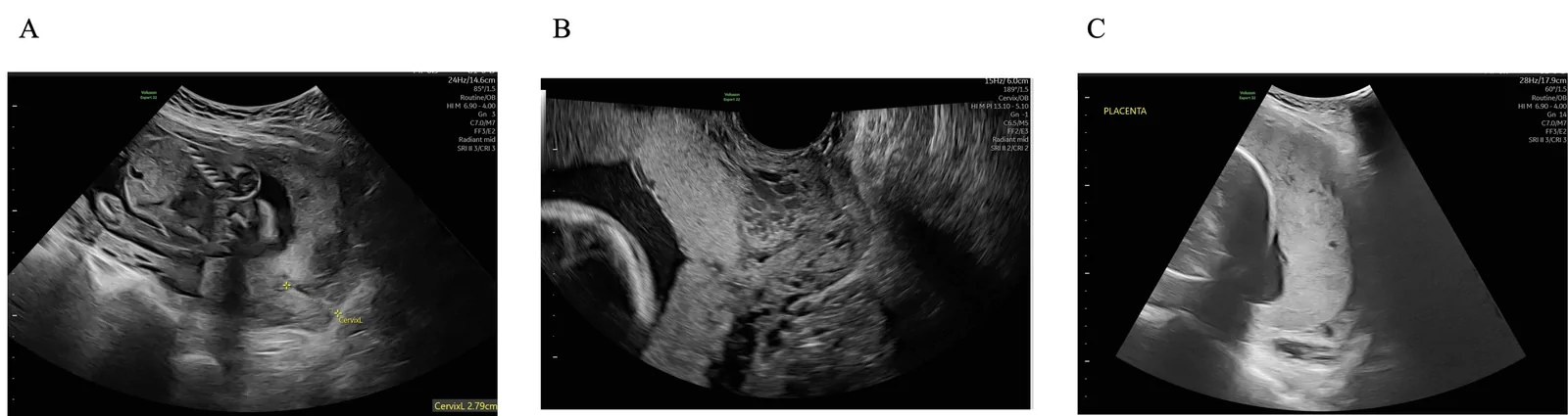
Prenatal Ultrasound Findings Demonstrating Persistent Complete Placenta Previa Without Sonographic Evidence of Placenta Accreta Spectrum (A) Transabdominal ultrasound obtained at 20 weeks demonstrating complete placenta previa without sonographic evidence of placenta accreta spectrum. The hypoechoic retroplacental space is clearly delineated, and the retroplacental myometrium is of normal thickness. (B) Transvaginal ultrasound obtained at 20 weeks again demonstrating complete placenta previa without placental lacunae. Color Doppler (not shown) did not demonstrate bridging vessels. (C) Follow-up transabdominal ultrasound obtained at 30 weeks demonstrating persistent placenta previa without overt sonographic findings of placenta accreta spectrum. The placental margin appears well defined, and no lacunae are identified.

Past medical history was significant for scoliosis, migraines, and polycystic ovarian syndrome. Obstetric history included three prior lower transverse cesarean deliveries and two prior dilation and curettage procedures. The patient was being routinely followed by maternal-fetal medicine (MFM) for known placenta previa (Figure 1a, 1b). An ultrasound, performed by MFM, two weeks prior to presentation (Figure 1c) demonstrated complete placenta previa with placental-myometrial borders appearing intact and no sonographic findings suggestive of PAS. The estimated fetal growth was at the 59th percentile with otherwise normal fetal anatomy and amniotic fluid volume. Planned delivery had been scheduled for 36-37 weeks of gestation.

Due to ongoing bleeding without evidence of hemorrhagic shock in the setting of placenta previa, the decision was made to proceed with emergent cesarean delivery. Preparations included administration of betamethasone for fetal lung maturation, notification of the neonatal intensive care unit (NICU), preparation of four units of packed red blood cells (pRBCs), and informed consent for possible cesarean hysterectomy. The patient received a routine preoperative one-liter bolus of lactated Ringer’s solution as well as intravenous ondansetron, metoclopramide, and famotidine for nausea prophylaxis. Spinal anesthesia was placed without complication at 0744, and surgical skin incision occurred at 0804.

Amniotomy was performed at 0820, delivery occurred at 0821, and the neonate required immediate resuscitation. Following delivery of the neonate, abnormal adherence of the placenta was identified, concerning for PAS. This was communicated to the anesthesia team and a plan was made for conversion to general anesthesia and for cesarean hysterectomy. Bleeding was minimal during this time. Shortly thereafter, the patient developed acute respiratory distress requiring endotracheal intubation at 0825. Cardiopulmonary resuscitation was initiated one minute later for pulseless electrical activity (PEA) arrest. The patient remained in PEA arrest during pulse checks at 0829, 0831, and 0834, with 1 mg epinephrine administered during each cycle. The return of spontaneous circulation was achieved at 0836 with restoration of organized rhythm and palpable pulse. Norepinephrine infusion was initiated for hemodynamic support.

Given the patient's risk factors, including known complete placenta previa, a history of three prior cesarean deliveries, the intraoperative finding of abnormal placental adherence concerning for PAS, and the abrupt onset of cardiopulmonary collapse with rapid development of coagulopathy, there was high clinical suspicion for AFE. Because AFE is a clinical diagnosis requiring immediate intervention, treatment was initiated based on the patient's presentation without awaiting confirmatory diagnostic studies. 

Massive transfusion protocol was activated during the resuscitation and postoperative period. With transfusions ongoing, CBC at 0927 showed hemoglobin at 6.1 g/dL, hematocrit at 20.2%, and platelet count at 75x10^9^/L. The patient ultimately received 22 units of pRBCs, 23 units of fresh frozen plasma, five units of platelets, and four units of cryoprecipitate. A supracervical hysterectomy was first completed while chest compressions were ongoing. Intraoperative chest radiography demonstrated diffuse bilateral patchy pulmonary opacities without effusions or pneumothorax.

Persistent hemorrhage and disseminated intravascular coagulopathy (DIC) complicated the remainder of the operation. There was continued massive hemorrhage from the remaining cervix requiring removal of the cervix. Once the remaining cervical tissue was removed, a bladder injury was identified after visualization of the Foley catheter within the pelvis. Urology was consulted intraoperatively; however, definitive bladder repair was deferred due to the patient’s critical instability with developing acute respiratory distress syndrome, hypothermia, and ongoing coagulopathy. Trauma surgery was subsequently consulted for abdominal and pelvic packing and assisted with placement of an AbThera wound vac. A plan was made for delayed primary closure following maternal stabilization. Total quantified blood loss during the operation was 12.1 liters. The patient was then transferred out of the operating room and into the intensive care unit at 1155 for continued resuscitation and management.

**Figure 2:**
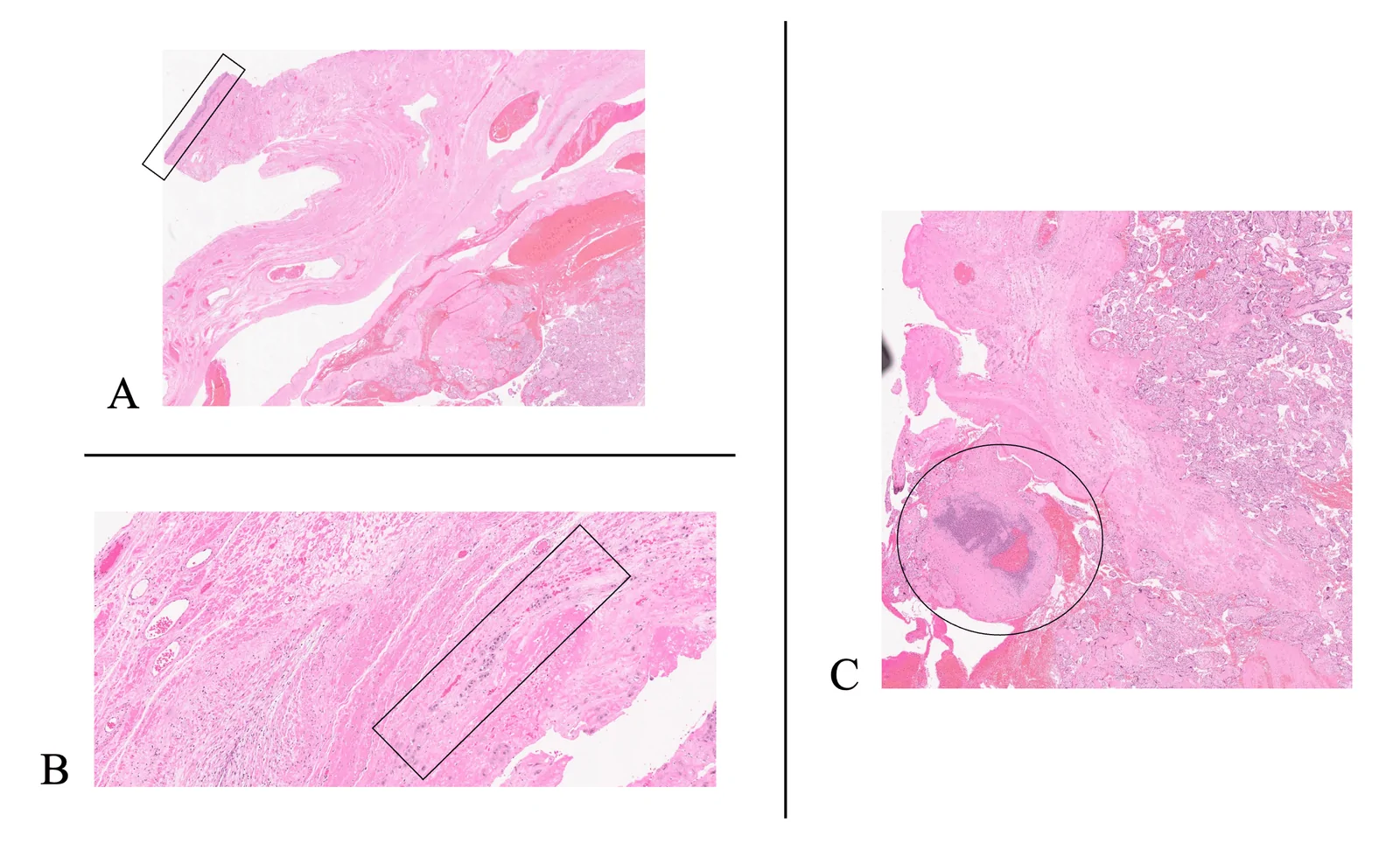
Histopathologic Findings Demonstrating Placenta Accreta Spectrum Grade 3D (A) Low-power hematoxylin and eosin (H&E, ×1) section of the anterior cervix demonstrating placental tissue extending deeply into the cervical stroma beneath the intact ectocervical epithelium. The rectangle highlights the intact ectocervical epithelium. (B) Higher-power H&E (×10) image demonstrating extravillous trophoblasts infiltrating the paracervical soft tissue, consistent with invasive placentation. The rectangle highlights the region of trophoblastic invasion. (C) H&E (×2) section demonstrating placental tissue extending through the cervical serosa, confirming placenta accreta spectrum grade 3D. The circle highlights the focus of placental tissue extending through the cervical serosa.

Transthoracic echocardiography performed on postoperative day one demonstrated normal left and right ventricular systolic function, mild tricuspid regurgitation, and no pericardial effusion, making primary cardiac etiologies and pulmonary vascular etiologies less likely. Final pathology, reported two days after the cesarean hysterectomy, demonstrated PAS grade 3D involving the anterior wall and lower uterine segment with greater than 75% myometrial invasion and 2.3 cm of serosal disruption (Figure 2a, 2b, 2c). Placental tissue additionally demonstrated remote infarcts.

In the intensive care unit, vasopressor support was successfully discontinued as hemodynamic stability improved. However, the patient's respiratory status worsened, with worsening acute respiratory distress syndrome requiring escalating ventilatory support and positive end-expiratory pressure of 12 cm H₂O. In consultation with maternal-fetal medicine specialists and a nearby tertiary care center, she was transferred approximately seven hours after cardiac arrest for higher-level critical care and consideration of extracorporeal membrane oxygenation (ECMO).

At the tertiary care center, on postoperative day one, the patient returned to the operating room for planned exploratory laparotomy and evaluation of suspected genitourinary injury by a multidisciplinary team including urology, maternal-fetal medicine, and gynecologic oncology. Intraoperatively, a significant bladder injury and bilateral ureteral injuries were identified. Given the extent of the urologic injuries and the patient's ongoing critical illness, definitive urinary reconstruction was deferred, and bilateral percutaneous nephrostomy tubes were placed for temporary urinary diversion. A right retroperitoneal hematoma was also identified, necessitating right salpingo-oophorectomy prior to abdominal closure.

ECMO was ultimately not required. The patient's respiratory status gradually improved, and she was successfully extubated on postoperative day three. She was discharged from the tertiary care center on postoperative day eight with bilateral nephrostomy tubes in place and plans for delayed definitive surgical management of her genitourinary injuries. The neonate remained stable throughout the hospitalization following admission to the neonatal intensive care unit.

Due to ongoing urologic sequelae from the bladder injury, the patient later underwent ileal conduit creation with Indiana pouch urinary diversion approximately three months after the initial hospitalization with an uncomplicated postoperative course. Despite substantial postoperative morbidity and prolonged recovery, both maternal and neonatal survival were ultimately achieved. A summary of the patient's clinical course is provided in Table 1.

**Table 1:** Timeline of Major Clinical Events

Event/Time	Clinical Event
Presentation	33 weeks and 1 day gestation with vaginal bleeding and contractions in the setting of complete placenta previa
Antepartum (2 weeks prior)	Ultrasound demonstrated complete placenta previa without evidence of placenta accreta spectrum
Preoperative course	Betamethasone, ondansetron, metoclopramide, and famotidine administered; neonatal intensive care unit notified; blood products prepared
0744	Spinal anesthesia placed
0804	Skin incision for cesarean delivery
0821	Delivery of neonate requiring resuscitation
0825	Acute respiratory distress requiring endotracheal intubation
0826	Pulseless electrical activity arrest; cardiopulmonary resuscitation initiated
0836	Return of spontaneous circulation achieved
Intraoperative course	Massive transfusion protocol activated; cesarean hysterectomy completed; persistent hemorrhage complicated by disseminated intravascular coagulation and bladder injury
ICU course	Acute respiratory distress syndrome requiring escalating ventilatory support
~7 hours after cardiac arrest	Transfer to tertiary care center for consideration of extracorporeal membrane oxygenation
Postoperative day 2	Pathology resulted: formalin specimen showed placenta accreta spectrum grade 3D with greater than 75% myometrial invasion and 2.3 cm of uterine serosal disruption; confirmed by two pathologists
Postoperative day 3	Successfully extubated
Postoperative day 8	Discharged with bilateral nephrostomy tubes
~3 months later	Ileal conduit creation with Indiana pouch urinary diversion without postoperative complications

## Discussion

AFE remains one of the most severe and unpredictable obstetric emergencies due to its abrupt presentation, rapid progression, and substantial maternal morbidity and mortality risks. Although the exact pathophysiology is not fully understood, current evidence supports a multifactorial inflammatory and immunologic process triggered by exposure of the maternal circulation to fetal or amniotic components [3,5]. Diagnosis remains clinical and one of exclusion, as no definitive confirmatory test exists and presentation frequently overlaps with other causes of maternal collapse including pulmonary embolism, anaphylaxis, septic shock, eclampsia, and obstetric hemorrhage [3,6]. Because treatment is time-sensitive, management should not be delayed while awaiting diagnostic studies. In the present case, the abrupt onset of respiratory failure, pulseless electrical activity arrest, and rapidly developing disseminated intravascular coagulopathy immediately following delivery prompted empiric treatment for AFE. Laboratory findings, including acute blood loss anemia and consumptive thrombocytopenia, were consistent with the rapidly developing disseminated intravascular coagulopathy observed clinically. Subsequent chest radiography demonstrated bilateral pulmonary infiltrates without edema or pneumothorax, while postoperative echocardiography demonstrated normal biventricular function. Together, these findings supported the clinical diagnosis of AFE while making primary cardiac and pulmonary etiologies of cardiac arrest and hemorrhage less likely. This case was further complicated by previously undiagnosed PAS with catastrophic hemorrhage, highlighting the diagnostic complexity of distinguishing AFE from other causes of maternal cardiovascular collapse and coagulopathy in the peripartum setting.

Recent population-level data suggest that PAS is among the strongest risk factors for AFE, with an adjusted odds ratio of 10.0 compared with pregnancies without PAS; the association appears to strengthen with increasing depth of placental invasion, with PAS grade 2 and 3A-D demonstrating an adjusted odds ratio of 17.4 for AFE [7]. In the present case, pathology demonstrated previously undiagnosed PAS grade 3D, raising the possibility that occult placental invasion contributed to the patient's risk for AFE despite the absence of prenatal imaging findings suggestive of PAS.

PAS is a pathologic diagnosis and therefore cannot be definitively confirmed or excluded by prenatal imaging alone. Clinical suspicion should instead be based on a combination of patient risk factors and imaging findings. The strongest risk factors include prior uterine surgery, particularly cesarean delivery, and placenta previa. Among patients with placenta previa, the risk of PAS increases to 3%, 11%, 40%, 61%, and 67% with zero, one, two, three, and four prior cesarean deliveries, respectively [8]. Given this patient's placenta previa and three prior cesarean deliveries, her estimated pretest risk of PAS was approximately 61%.

Ultrasound findings suggestive of PAS include placental vascular lacunae, loss of the retroplacental hypoechoic zone, myometrial thinning, bridging vessels, and disruption of the uterine-bladder interface [9]. None of these findings were identified prenatally in this case. However, the reported sensitivity of ultrasound for PAS is approximately 87%-94% [10], indicating that a normal prenatal ultrasound cannot reliably exclude PAS, particularly in patients with substantial baseline risk.

For patients at an elevated risk of PAS based on clinical risk factors, imaging findings, or both, delivery at a tertiary care center with multidisciplinary planning is recommended and has been associated with improved maternal outcomes [9]. Nevertheless, because a proportion of PAS cases remain undiagnosed until delivery, all hospitals providing obstetric care should maintain contingency plans for unexpected PAS. These plans should include rapid mobilization of experienced surgical personnel, activation of massive transfusion protocols, and established pathways for escalation or transfer to higher levels of care when appropriate.

Historically, AFE was associated with maternal mortality rates exceeding 60%; however, outcomes have improved substantially with advances in obstetric critical care, massive transfusion protocols, prompt cardiopulmonary resuscitation, and coordinated multidisciplinary management [4,11]. Early recognition and prompt mobilization of obstetric, anesthesia, nursing, blood bank, and critical care teams are essential to optimizing maternal and neonatal outcomes. DIC develops rapidly in many patients with AFE and contributes significantly to morbidity through severe hemorrhage and consumptive coagulopathy [4]. Prompt correction of coagulopathy with blood product replacement, particularly fibrinogen replacement, remains a cornerstone of management. In this case, rapid multidisciplinary intervention, aggressive supportive resuscitation, and correction of coagulopathy were associated with favorable maternal and neonatal outcomes despite the severity of presentation.

Emerging adjunctive therapies for AFE, including the proposed atropine, ondansetron, and ketorolac (AOK) regimen, have been described in case reports and small observational studies; however, evidence supporting these interventions remains limited [12]. Although the AOK regimen has been proposed as a potential treatment strategy targeting the hypothesized inflammatory and vasoactive components of AFE, no standardized recommendations currently exist regarding its routine use. In the present case, maternal and neonatal survival were achieved without the administration of the complete AOK regimen, emphasizing that early recognition, supportive resuscitation, hemodynamic stabilization, and correction of coagulopathy remain the foundation of management. Given the rarity of AFE and the limited prospective data available, additional research is needed to better define the role of adjunctive therapies in improving outcomes.

This case highlights the importance of maintaining a high index of suspicion for AFE in the setting of sudden maternal cardiopulmonary collapse, particularly among patients with PAS. Given the unpredictable and rapidly progressive nature of AFE, preparedness and standardized multidisciplinary response systems are essential. Simulation-based training and institution-specific response protocols may improve team communication, expedite intervention, and optimize patient outcomes [13].

## Conclusions

AFE remains a rare and catastrophic obstetric emergency requiring rapid recognition, aggressive supportive resuscitation using current standardized protocols, and coordinated multidisciplinary management. This case emphasizes the diagnostic complexity of AFE occurring concurrently with previously undiagnosed PAS and catastrophic obstetric hemorrhage. Despite prolonged PEA arrest, DIC, massive transfusion requirements, and substantial postoperative morbidity, both maternal and neonatal survival were ultimately achieved.

## References

[1] Carlson K, Mikes BA (2024). Amniotic fluid embolism. StatPearls.

[2] Fong A, Chau CT, Pan D, Ogunyemi DA (2015). Amniotic fluid embolism: antepartum, intrapartum and demographic factors. J Matern Fetal Neonatal Med.

[3] Clark SL, Romero R, Dildy GA (2016). Proposed diagnostic criteria for the case definition of amniotic fluid embolism in research studies. Am J Obstet Gynecol.

[4] Pacheco LD, Saade G, Hankins GD, Clark SL (2016). Amniotic fluid embolism: diagnosis and management. Am J Obstet Gynecol.

[5] Suvannasarn R, Tongsong T, Jatavan P (2020). Amniotic fluid embolism: the pathophysiology, diagnostic clue, and blood biomarkers indicator for disease prediction. Clin Exp Obstet Gynecol.

[6] Stafford IA, Moaddab A, Dildy GA, Klassen M, Belfort MA, Romero R, Clark SL (2019). Evaluation of proposed criteria for research reporting of amniotic fluid embolism. Am J Obstet Gynecol.

[7] Mazza GR, Youssefzadeh AC, Klar M (2022). Association of pregnancy characteristics and maternal outcomes with amniotic fluid embolism. JAMA Netw Open.

[8] Silver RM, Landon MB, Rouse DJ (2006). Maternal morbidity associated with multiple repeat cesarean deliveries. Obstet Gynecol.

[9] (2018). Obstetric Care Consensus No. 7: Placenta Accreta Spectrum. Obstet Gynecol.

[10] D'Antonio F, Iacovella C, Bhide A (2013). Prenatal identification of invasive placentation using ultrasound: systematic review and meta-analysis. Ultrasound Obstet Gynecol.

[11] Conde-Agudelo A, Romero R (2009). Amniotic fluid embolism: an evidence-based review. Am J Obstet Gynecol.

[12] Pacheco LD, Clark SM, Fox K, Bauer ME, Clark SL (2026). Use of atropine, ondansetron, and ketorolac in suspected amniotic fluid embolism. Obstet Gynecol.

[13] Combs CA, Montgomery DM, Toner LE, Dildy GA (2021). Society for Maternal-Fetal Medicine Special Statement: checklist for initial management of amniotic fluid embolism. Am J Obstet Gynecol.

